# Clinical nursing mentors’ motivation, attitude, and practice for mentoring and factors associated with them

**DOI:** 10.1186/s12912-024-01757-8

**Published:** 2024-01-30

**Authors:** Yan Wang, Suzhen Hu, Jiali Yao, Yangmiao Pan, Junling Wang, Hua Wang

**Affiliations:** 1 Nursing Institute, Ningbo College of Health Science, Ningbo, 315100 P.R. China; 2 Delivery and Labor Room, Zhejiang University Mingzhou Hospital, Ningbo, 315100 P.R. China; 3 Department of Infectious Diseases, Ningbo No.2 Hospital, Ningbo, 315000 P.R. China; 4 Department of Obstetrics, Affiliated Hangzhou First People’s Hospital, Zhejiang University School of Medicine, Hangzhou, 310006 P.R. China; 5 Nursing department, Zhejiang University Mingzhou Hospital, Ningbo, 315100 P.R. China

**Keywords:** Clinical nursing mentor, Teaching motivation, Attitude, Practice, Cross-sectional study

## Abstract

**Objective:**

To investigate the motivation, attitude, and practice toward mentoring and related factors among clinical nursing mentors.

**Methods:**

This cross-sectional study included clinical nursing mentors from 30 hospitals in Zhejiang Province between August and September 2023. Demographic information, motivation, attitude, and practice were collected through a self-administered questionnaire.

**Results:**

A total of 495 valid questionnaires were collected, and most of the participants were 30–39 years old (68.7%). Average motivation, attitude, and practice scores were 29 [26, 32] (possible range: 8–40), 87 (82, 94) (possible range: 22–110), and 41 (38, 45) (possible range: 11–55), respectively. Correlation analyses showed that the motivation scores were positively correlated with attitude scores (*r* = 0.498, *P* < 0.001) and practice scores (*r* = 0.408, *P* = 0.001), while attitude scores were positively correlated with practice scores (*r* = 0.554, *P* < 0.001). Multivariate logistic regression showed that intermediate and senior nursing mentors (OR = 0.638, 95% CI: [0.426–0.956], *P* = 0.030) and different hospitals (OR = 1.627, 95% CI: [1.054–2.511], *P* = 0.028) were independently associated with motivation. The hospital’s frequency of psychological care was a significant factor associated with nursing mentoring motivation, attitude, and practice. Participation in training (OR = 2.908, 95% CI: [1.430, 5.913], *P* = 0.003) and lower frequency of job evaluation in hospital (“Often”: OR = 0.416, 95% CI: [0.244–0.709], *P* = 0.001 and “Sometimes”: OR = 0.346, 95% CI: [0.184–0.650], *P* = 0.001) were independently associated with practice.

**Conclusion:**

Clinical nursing mentors had adequate motivation, positive attitude, and proactive practice towards mentoring and associated factors. Clinical nursing mentorship should be enhanced by prioritizing mentor training, fostering a supportive environment with consistent psychological care, and promoting structured mentorship activities.

**Supplementary Information:**

The online version contains supplementary material available at 10.1186/s12912-024-01757-8.

## Background

By assisting nursing students through inquiry and offering guidance and feedback on patient-centered clinical learning, faculty members have a vital role in fostering their development and achievement in extracurricular activities beyond traditional classroom settings [1]. These extracurricular activities serve as a gateway through which faculty can introduce students to various clinical practices, nursing research, educational experiences, and service-related opportunities, including tutoring and committee involvement [2, 3]. However, the existing approach to assigning mentoring responsibilities in China predominantly relies on objective criteria, such as qualifications, skills, and organizational considerations, with limited emphasis on the mentor’s motivation and willingness, which may inadvertently lead to mentors not fully engaging in their roles, suboptimal mentoring outcomes and potential nurse attrition concerns [4].

In order to gain a deeper understanding of the mentors’ motivation, attitudes, and practices, it is essential to consider various psychological and sociological theories. The Psychological Needs Motivation Theory posits that individual behaviors are typically influenced by intrinsic and extrinsic motivations [4], allowing us to gain insights into how mentors’ internal needs impact their willingness to assume mentoring responsibilities. On the other hand, Social Exchange Theory asserts that an individual’s social behaviors are shaped by economic and social exchanges [5]. This theory aids in explaining the dynamics of interactions and relationships between mentors and apprentices and how these factors influence the mentor’s attitudes and behaviors. The Theory of Planned Behavior focuses on individual decision-making processes and can be applied to analyze the mentor’s thought process when making mentoring-related decisions [6]. These psychological and sociological theories have a significant role in comprehensively understanding and elucidating mentors’ motivation, attitudes, and practices.

Individual willingness is pivotal for harnessing an individual’s subjective initiative and enhancing the quality of mentoring [7, 8]. Therefore, a profound understanding of mentors’ willingness and influencing factors is crucial for enhancing the effectiveness of mentoring. This study explored the motivation, attitude, and practice of clinical nursing mentors and the factors associated with these aspects. The ultimate objective was to help organizations establish motivation mechanisms for nursing mentors, which, in turn, could foster their enthusiasm for mentoring, enhance the effectiveness of nurse apprenticeship programs, and provide practical insights for nursing human resource development and the advancement of the nursing profession.

## Methods

### Study design and participants

This cross-sectional survey included clinical nursing mentors from 30 hospitals in Zhejiang Province between August and September 2023. Inclusion criteria were the following: (1) the mandatory qualifications to practice as a nurse; (2) competent in mentoring newly recruited or practical nurses; (3) used to mentor new nurses/interns nurses; and (4) willingly consented to participate in the survey study. Exclusion criteria were the following: (1) submission of incomplete information; (2) selection of identical options for an entire section of the questionnaire; (3) completion of the questionnaire in < 120 s or > 60 min; (4) no experience as a clinical nursing mentor; and (5) instances of duplicate IP data.

The study was ethically approved by the Medical Ethics Committee of Ningbo College of Health Sciences. Before participating, all participants were provided detailed information about the study’s purpose and content and signed an informed consent. Emphasis was placed on the confidentiality of their responses, assuring them that their personal data would be securely handled and used exclusively for research purposes. Informed consent was obtained from each participant, ensuring that only those who understood and agreed to these terms were included in the analysis.

### Questionnaire introduction

The questionnaire was designed following established guidelines and pertinent literature. Subsequently, feedback was solicited from a panel of 10 senior clinical nursing and nursing education experts, encompassing individuals holding professional titles ranging from Chief Nursing Officer to Associate Professors and Professors. Their expert insights were used to effectuate refinements and incorporate their feedback into the questionnaire. These revisions involved enhancements to question wording and options to ensure better alignment with real-world clinical scenarios.

The experts suggested including more relevant elements to further elucidate the factors affecting mentoring motivation. These elements were the following: [1] whether hospitals offered training on teaching skills for mentors, [2] how the hospital evaluated mentoring work, and [3] the impact of mentoring apprentices on the mentor’s nursing duties. These recommendations were carefully incorporated to enhance the comprehensiveness and relevance of the questionnaire.

The questionnaire was subsequently administered in a single small-scale distribution, yielding 48 completed copies and demonstrating robust internal consistency with a Cronbach’s α coefficient of 0.904. The reliability of different dimensions was also strong: Cronbach’s α coefficient of motivation, attitude, and practice dimension were 0.833, 0.873, and 0.616, respectively, indicating good internal consistency across all sections. As a result, no adjustments were made to the questionnaire. The final questionnaire encompassed four dimensions, i.e., demographic information (including education, gender, institutional nature, professional title, and institutional support for mentoring work), motivation dimension, attitude dimension, and practice dimension, totaling 61 questions.

In response to expert feedback, modifications were made to enhance the clarity and precision of the questionnaire. First, terms such as ‘newcomer’, ‘new nurse’, and ‘apprentice’ were standardized to ‘new nurse/intern nurse’ throughout the questionnaire. This change ensured consistency and clarity. Second, certain question stems were rephrased for better accuracy. For example, the phrase ‘the extent of the hospital’s psychological care for mentoring nurses’ was altered to ‘the frequency of the hospital’s psychological care for mentoring nurses’. This adjustment provided a more direct measure of the hospital’s support in this area. Additionally, at the beginning of the questionnaire, a clear definition of a ‘nursing mentor’ was introduced, which served to screen participants for the study accurately. Participants were asked to confirm whether they currently serve or have previously served as clinical nursing mentors. Furthermore, two open-ended questions were added to gain deeper insights into the mentors’ perspectives. These questions revolve around the mentors’ comprehensive evaluation of their apprentices, specifically asking about aspects they deemed most and least important: practical skills, communication skills, work attitude, learning ability, or innovative spirit. These additions aimed to explore the priorities and values of mentors in their mentorship roles, thus offering a nuanced understanding of their approach to mentorship.

The motivation dimension comprised 8 questions, each evaluated on a five-point Likert scale ranging from strongly disagree [1] to strongly agree [5], resulting in a score range of 8–40. The attitude dimension consisted of 22 questions, evaluated on a five-point Likert scale with scores ranging from strongly disagree [1] to strongly agree [5], yielding a score range of 22–110. The practice dimension featured 11 questions, evaluated on a five-point Likert scale with response options ranging from always [5] to never [1], and scores ranging from 11 to 55. Notably, three practice questions were open-ended and not assigned numerical scores. Scores > 70% of the maximum in each section indicated adequate motivation, positive attitude, and proactive practice [9].

The formal experimental data analysis revealed strong internal consistency with a Cronbach’s α coefficient of 0.883 and a Kaiser-Meyer-Olkin (KMO) value of 0.918, confirming the reliability and suitability of the questionnaire for the research study.

The sample size for our study was calculated using a standard statistical formula. With a confidence level of 95% (z = 1.96), an estimated proportion (p) of 0.5, and a margin of error (e) of 0.05, the formula n = z^2^ * p * (1 - p) / e^2^ yielded a sample size of approximately 384 [10]. However, to account for potentially unusable responses, a non-response rate of 10% was considered, and we aimed to collect > 500 questionnaires. This study employed a convenient sampling approach to select 30 hospitals in 8 cities within Zhejiang province. The hospitals were chosen based on their qualification for clinical internship mentoring and their minimum classification at the secondary level. The distribution of questionnaires to clinical nursing mentors within these hospitals was facilitated through the hospital’s nursing department clinical practice management personnel, utilizing the WeChat platform. There was an estimated pool of approximately 5,000 internship mentors among the selected hospitals. The allocation of questionnaires was proportional to the number of hospital beds. Hospitals with < 1,000 beds received approximately 5–10 questionnaires, those with 1,000–2,000 beds were assigned 10–20 questionnaires, and hospitals with > 2,000 beds received 20–50 questionnaires. A total of 643 questionnaires were distributed, with 25 individuals declining to participate, resulting in 618 collected questionnaires. After excluding 37 questionnaires with a completion time of < 120 s or > 3,600 s, 82 questionnaires that were completed by individuals who had never served as clinical nursing mentors, and 4 questionnaires with repeated IP addresses, a total of 495 valid questionnaires were included in the statistical analysis.

### Statistical analysis

Statistical analysis was conducted using SPSS 23. The respondents’ demographic information and their scores across various dimensions were subjected to descriptive analysis. The median, 25th percentile, and 75th percentile were used to present these data. For different demographic characteristics, count data was represented as N (%). In terms of comparing the differences in dimension scores among survey participants with varying demographic characteristics, the Wilcoxon-Mann-Whitney test was employed for comparisons between the two groups. For continuous variables across three or more groups, the Kruskal-Wallis variance analysis method was used. The Spearman correlation coefficient was applied to analyze the correlation between scores across different dimensions. In both univariate and multivariate regression analyses, dimension scores were used as dependent variables to analyze their relationship with demographic data. In multivariate analysis, the median score was used as the cut-off value. A stepwise approach was adopted for selecting model variables. Variables with a significance level of *P* < 0.1 in univariate analysis were initially included. In this analysis, *P*-values were retained to three decimal places, with *P* < 0.05 representing statistical significance.

## Results

### The general characteristics of the participants

A total of 495 valid questionnaires were collected. Among them, most participants were 30–39 years old (68.7%); 98% were female, 92.9% had undergraduate or higher educational levels, and 68.1% held professional titles at an intermediate and senior level. The participants had varying years of nursing experience, with the majority falling within the 6–15 years (66.7%). There were 65% who served as nursing supervisors for 3–10 years, and over half of the nursing mentors had guided more than 11 apprentices. Also, 74.3% came from Tertiary A hospitals, 84.8% became nursing mentors through organizational arrangements, and 81.4% had undergone training related to mentoring (Table 1).

**Table 1:** Participants’ baseline information and distribution of motivation, attitude, and practice scores

	N(%)	Motivation			Attitude			Practice	
		Median (25% quartile, 75% quartile)	*P*		Median (25% quartile, 75% quartile)	*P*		Median (25% quartile, 75% quartile)	*P*
**Total**	495	29(26, 32)			87(82, 94)			41(38, 45)	
**Age_adjusted**			0.959			0.141			0.374
20–29 years	64(12.9)	29(27, 31)			84.5(81, 91.5)			40(37, 44)	
30–39 years	340(68.7)	29(26, 32)			87(82, 94)			41(38, 45)	
≥ 40 years	91(18.4)	28(27, 32)			89(82, 96)			41(38, 45)	
**Gender**			0.175			0.736			0.201
Male	10(2.0)	26.5(24, 31)			85.5(82, 96)			39(36, 43)	
Female	485(98.0)	29(26, 32)			87(82, 94)			41(38, 45)	
**Education**			0.604			0.826			0.850
Junior college and below	35(7.1)	29(26, 33)			87(81, 95)			41(37, 46)	
Undergraduate	453(91.5)	29(26, 31)			87(82, 94)			41(38, 45)	
Postgraduate	7(1.4)	28(22, 32)			86(82, 88)			40(36, 45)	
PhD and above	0(0)	/			/			/	
**Education_adjusted**			0.468			0.840			0.754
Junior college and below	35(7.1)	29(26, 33)			87(81, 95)			41(37, 46)	
Undergraduate and above	460(92.9)	29(26, 31)			87(82, 94)			41(38, 45)	
**Professional title_adjusted**			0.035			0.126			0.940
Junior (Nurse/Nurse Practitioner)	158(31.9)	30(27, 32)			86(81, 93)			41(38, 45)	
Intermediate and Senior (Nurse Practitioner-in-Charge/Deputy Chief Nurse/Chief Nurse)	337(68.1)	28(26, 31)			87(82, 95)			41(38, 45)	
**Years of nursing experience**			0.634			0.003			0.526
≤ 5 years	30(6.1)	29.5(27, 31)			85.5(81, 92)			40.5(37, 44)	
6–10 years	145(29.3)	28(26, 32)			85(81, 90)			40(38, 44)	
11–15 years	185(37.4)	29(26, 32)			89(83, 96)			41(38, 45)	
16–20 years	96(19.4)	28(26, 31)			86.5(81, 95.5)			41(38, 45.5)	
≥ 21	39(7.9)	29(27, 32)			91(83, 95)			41(38, 45)	
**Grade of hospital_adjusted**			0.120			0.325			0.330
Tertiary A	368(74.3)	28.5(26, 31)			87(82, 95)			41(38, 45)	
Other	127(25.7)	29(27, 32)			86(81, 93)			41(38, 45)	
**Years of nursing supervision_adjusted**			0.360			0.034			0.567
< 1 year	30(6.1)	30(27, 32)			88.5(83, 96)			41(38, 45)	
1–2 years	46(9.3)	28.5(26, 31)			85.5(81, 92)			40.5(38, 44)	
3–5 years	167(33.7)	29(26, 32)			86(81, 93)			41(38, 45)	
6–10 years	155(31.3)	29(26, 32)			88(82, 96)			41(38, 44)	
11–15 years	69(13.9)	28(25, 31)			87(82, 96)			41(38, 45)	
≥ 15 years	28(5.7)	29(26.5, 30.5)			91(84.5, 99)			42.5(39, 46)	
**Number of persons mentored**			0.748			0.033			0.564
1–2	39(7.9)	28(27, 31)			86(82, 93)			40(37, 45)	
3–5	82(16.6)	29(26, 32)			85(80, 93)			40(37, 44)	
6–10	93(18.8)	29(26, 31)			86(82, 92)			41(39, 44)	
≥ 11	281(56.8)	29(26, 32)			88(82, 96)			41(38, 45)	
**Pathway to becoming a mentor_adjusted**			0.033			0.485			0.112
Self-application	61(12.3)	30(27, 32)			88(83, 96)			42(40, 45)	
Organization arrangement	420(84.8)	28.5(26, 31)			87(82, 94)			41(38, 45)	
Other	14(2.8)	30(27, 33)			83.5(79, 93)			40.5(38, 43)	
**Training attending**			0.006			0.004			<0.001
Have not attended training	56(11.3)	27(25, 29)			84(78, 90.5)			38(34.5, 40)	
Did attend training	403(81.4)	29(27, 32)			87(82, 95)			41(39, 45)	
Don’t remember	36(7.3)	28(24.5, 31)			86.5(80, 92.5)			39(36.5, 44.5)	
**Salary subsidies granted by hospital**			<0.001			0.006			<0.001
Always	86(17.4)	30(27, 33)			91(83, 98)			44(39, 47)	
Often	64(12.9)	30(28, 33)			88(83, 94.5)			41.5(39, 45)	
Sometimes	76(15.4)	29(27.5, 32)			86.5(81, 93)			41(37, 44)	
Rarely	70(14.1)	29(26, 32)			87(82, 93)			41(38, 45)	
Hardly ever	199(40.2)	28(25, 30)			85(81, 93)			40(37, 44)	
**Degree of hospital financial support**			<0.001			<0.001			<0.001
Adequate	115(23.2)	31(28, 33)			91(83, 99)			44(40, 47)	
Much	71(14.3)	30(28, 32)			87(82, 91)			41(39, 45)	
General	175(35.4)	28(26, 31)			86(81, 92)			41(38, 44)	
Very little	48(9.7)	28(26.5, 32)			87(81.5, 92)			39(37, 42.5)	
Hardly ever	86(17.4)	27(25, 29)			85(81, 93)			39.5(36, 43)	
**Frequency of psychological care performed by hospital**			<0.001			<0.001			<0.001
Always	126(25.5)	31(28, 33)			93(85, 99)			44(41, 47)	
Often	138(27.9)	29.5(27, 32)			88(82, 94)			41(39, 45)	
Sometimes	144(29.1)	28(26, 30)			85(81, 90)			40(37, 43)	
Rarely	61(12.3)	28(26, 30)			84(80, 91)			38(36, 41)	
Hardly ever	26(5.3)	25(23, 27)			82(75, 85)			35.5(34, 40)	
**Frequency of hospital to understanding issues in mentoring**			0.065			<0.001			<0.001
Always	173(34.9)	30(27, 32)			91(84, 98)			44(40, 47)	
Often	172(34.7)	28(26, 31)			86(82, 93)			40(38, 43.5)	
Sometimes	98(19.8)	29(27, 31)			85(80, 90)			39(36, 42)	
Rarely	36(7.3)	28(26, 31)			83(78.5, 90.5)			40(38, 43.5)	
Hardly ever	16(3.2)	28.5(26, 30.5)			82(78, 89.5)			39(35.5, 41)	
**Frequency of job evaluation in hospital**			<0.001			<0.001			<0.001
Always	136(27.5)	31(27.5, 33)			91(83.5, 98)			45(40.5, 47)	
Often	232(46.9)	29(26, 31)			86(82, 93)			40(38, 44)	
Sometimes	110(22.2)	28(26, 30)			85(81, 91)			39(36, 41)	
Rarely	10(2.0)	26.5(25, 32)			83.5(76, 90)			38.5(35, 39)	
Hardly ever	7(1.4)	23(19, 30)			77(70, 92)			38(34, 42)	

### Motivation, attitude, and practice scores and distribution across different populations

The research population had an average motivation score of 29/40 [26, 32], an attitude score of 87/110 (82, 94), and an average practice score of 41/55 (38, 45). Nursing mentors with a primary professional title had significantly higher motivation scores than those with intermediate or advanced professional titles (*P* = 0.035). Significant differences in attitude scores were observed among nursing mentors with varying years of nursing experience, years of nursing supervision, and the number of apprentices they guided (*P* < 0.05). Differences in motivation scores were statistically significant among nursing mentors based on the pathways they took to become mentors (*P* = 0.033). Participation in training and the frequency of hospitals providing compensation subsidies to nursing mentors significantly differed among groups regarding mentoring motivation, attitude, and practice (*P* < 0.01). The level of financial support from hospitals for nursing mentoring, the frequency of psychological care provided by hospitals to nursing mentors, and the frequency at which hospitals were aware of mentoring issues significantly differed in mentoring motivation, attitude, and practice scores (*P* < 0.001) (Table 1).

In terms of their motivation for mentoring, a considerable 37.8% expressed uncertainty regarding whether it was driven by aspirations for professional advancement (M1). Interestingly, 64.6% strongly disagreed or expressed disagreement with the idea that it was primarily for financial gain (M2). A noTable 36.6% indicated that their mentoring involvement was spurred by organizational assignments (M3). Impressively, a significant 86.7% perceived new nurses/interns not merely as apprentices but as collaborative working partners (M4). An overwhelming 95.5% affirmed that their motivation was rooted in facilitating the swift competence development of newcomers in the nursing field (M5). Furthermore, 62% were motivated by a desire to delve into the intricacies of nursing workforce development (M6). A striking 90.3% named their commitment to preserving the essence of nursing professionalism as a pivotal motivation (M7). Moreover, 83.5% expressed a noble intention to instill a passion for the profession in the hearts of the novices (Table 2).

**Table 2:** Responses to motivation dimension

	Strongly agree	Agree	Unsure	Disagree	Strongly disagree
1. I mentor new nurses/interns to accumulate opportunities for promotion.	48(9.7)	110(22.2)	187(37.8)	91(18.4)	59(11.9)
2. Participating in mentoring new nurses/interns is a way for me to earn more financial rewards.	19(3.8)	45(9.1)	111(22.4)	170(34.3)	150(30.3)
3. I guide new nurses/interns because it is an organizational assignment, and I have no choice.	48(9.7)	133(26.9)	140(28.3)	138(27.9)	36(7.3)
4. When mentoring new nurses/interns, I prefer to see them as partners in our work rather than in a mentor-apprentice relationship.	190(38.4)	239(48.3)	50(10.1)	15(3.0)	1(0.2)
5. I guide new nurses/interns to help them become competent in nursing quickly.	256(51.7)	217(43.8)	18(3.6)	4(0.8)	0(0)
6. I mentor new nurses/interns to study the patterns of nursing manpower development.	107(21.6)	200(40.4)	134(27.1)	45(9.1)	9(1.8)
7. I guide new nurses/interns to pass on the spirit of the nursing profession.	188(38.0)	259(52.3)	39(7.9)	6(1.2)	3(0.6)
8. I guide new nurses/interns to make them fall in love with the noble nursing profession.	175(35.4)	238(48.1)	69(13.9)	6(1.2)	7(1.4)

The participants displayed diverse attitudes, with 43.6% either not perceiving or being uncertain about the personal benefits of mentoring new nurses/interns (A2). Interestingly, 19% viewed mentoring as time-consuming (A3), and an equal proportion found reporting and recording procedures cumbersome, which somewhat diminished their enthusiasm for mentoring (A4). On a positive note, 57.2% believed that mentoring could alleviate their workload through the contributions of newcomers (A5). Additionally, a respective 89.5% and 94% derived happiness (A13) and a sense of being valued (A14) from the growth of the mentees. Impressively, 96.7% expressed a keen interest in sharing their accumulated experiences and lessons with the younger generations (A19). Another significant majority, i.e., 95.5% (A20), displayed genuine concern for new nurses/interns, drawing from their past experiences (Table 3).

**Table 3:** Responses to attitude dimension

	Strongly agree	Agree	Unsure	Disagree	Strongly disagree
1.1 The nursing mentor system can help coordinate issues related to the scheduling of new nurses/interns. P	161(32.5)	251(50.7)	64(12.9)	8(1.6)	11(2.2)
1.2 The nursing mentor system can help balance the work and family life of new nurses/interns P	105(21.2)	213(43.0)	143(28.9)	21(4.2)	13(2.6)
1.3 The nursing mentor system can help coordinate relationships between new nurses/interns and colleagues. P	145(29.3)	306(61.8)	39(7.9)	2(0.4)	3(0.6)
2. Although participating in guiding new nurses, the growth of new nurses/interns is beneficial to themselves, but it doesn’t benefit me much. N	41(8.3)	59(11.9)	116(23.4)	226(45.7)	53(10.7)
3. Participating in mentoring new nurses/interns takes up too much of my time. N	25(5.1)	69(13.9)	161(32.5)	196(39.6)	44(8.9)
4. The reporting and documentation tasks during the guidance of new nurses/interns are cumbersome and affect my enthusiasm for mentoring. N	26(5.3)	68(13.7)	133(26.9)	211(42.6)	57(11.5)
5. Participating in mentoring new nurses/interns allows them to share some of my workload. P	45(9.1)	238(48.1)	126(25.5)	75(15.2)	11(2.2)
6. Participating in mentoring new nurses/interns is beneficial for building a good relationship with the nursing department (superiors). P	54(10.9)	142(28.7)	155(31.3)	112(22.6)	32(6.5)
7. Participating in mentoring new nurses/interns can help discover talent and build a broader network of relationships. P	79(16.0)	217(43.8)	132(26.7)	57(11.5)	10(2.0)
8. Mentoring new nurses/interns pushes me to continue learning. P	130(26.3)	297(60.0)	48(9.7)	13(2.6)	7(1.4)
9. Being able to learn new ideas and concepts from young nurses is very helpful to me. P	160(32.3)	290(58.6)	42(8.5)	2(0.4)	1(0.2)
10. Participating in mentoring new nurses/interns is helpful for me to learn new knowledge and experiences from other mentors. P	154(31.1)	294(59.4)	42(8.5)	5(1.0)	0(0)
11. I also received help from others when I was newly employed, so I am willing to actively mentor new nurses/interns. P	200(40.4)	269(54.3)	25(5.1)	1(0.2)	0(0)
12. Participating in mentoring new nurses/interns can earn me recognition and approval from colleagues. P	120(24.2)	257(51.9)	96(19.4)	21(4.2)	1(0.2)
13. Helping new nurses/interns grow makes me feel very happy. P	166(33.5)	277(56.0)	49(9.9)	3(0.6)	0(0)
14. Watching new nurses/interns grow under my guidance makes me feel my own value. P	184(37.2)	281(56.8)	26(5.3)	4(0.8)	0(0)
15. New mentors should receive training on educational and teaching abilities. P	199(40.2)	278(56.2)	15(3.0)	3(0.6)	0(0)
16. Nurses should only take on mentoring tasks after a comprehensive assessment. P	192(38.8)	279(56.4)	23(4.6)	1(0.2)	0(0)
17. I will guide new nurses/interns more if allowed to become an excellent mentor. P	181(36.6)	283(57.2)	24(4.8)	7(1.4)	0(0)
18. The organization has nurtured me, and I should also nurture new talents for the organization. P	188(38.0)	276(55.8)	27(5.5)	4(0.8)	0(0)
19. Serving as a mentor allows me to share my experiences and lessons with youngsters, helping them avoid unnecessary mistakes. P	220(44.4)	259(52.3)	15(3.0)	1(0.2)	0(0)
20. I care deeply about new nurses/interns because I have had similar experiences. P	206(41.6)	267(53.9)	20(4.0)	2(0.4)	0(0)

Regarding practice, 87.0% of respondents consistently and frequently adapted their mentoring approaches based on the distinct personalities of new nurses/interns (P1). Furthermore, 82.6% affirmed that the mentoring process compelled them to continually enhance their nursing knowledge and overall competence to varying degrees (P5), with a commendable 33.9% never contemplating giving up (P6). Surprisingly, 42.0% never selected mentees based on personal preferences (P7), and 43.2% maintained patience, even in the face of repeated poor performance by the newcomer (P8). Significantly, 73.8% advocated exposing the newcomer to a higher frequency of clinical practice (P10). A noteworthy 48.1% reported encountering conflicts between their mentoring responsibilities and clinical duties (P11). Regarding the overall evaluation of mentored new nurses/interns, 57.4% regarded work attitude as the most crucial factor (P13), whereas 67.3% deemed a creative spirit as less essential (P14) (Table 4).

**Table 4:** Responses to practice dimension

	Always	Often	Sometimes	Rarely	Hardly ever
1. I will adjust the guidance methods for new nurses/interns with different personalities. P	218(44.0)	213(43.0)	62(12.5)	2(0.4)	0(0)
2. I care about the learning, work, and life issues encountered by new nurses/interns. P	137(27.7)	213(43.0)	113(22.8)	16(3.2)	16(3.2)
3. I regularly summarize methods and organize records during the guidance of new nurses/interns. P	131(26.5)	189(38.2)	143(28.9)	30(6.1)	2(0.4)
4. When I encounter problems that cannot be resolved during the guidance of new nurses/interns, I seek help from the department or nursing department. P	105(21.2)	139(28.1)	188(38.0)	47(9.5)	16(3.2)
5. I continuously enhance my nursing knowledge and comprehensive abilities during the guidance of new nurses/interns. P	172(34.7)	237(47.9)	83(16.8)	3(0.6)	0(0)
6. The frequency with which I consider giving up while mentoring new nurses/interns. N	29(5.9)	34(6.9)	87(17.6)	177(35.8)	168(33.9)
7. If conditions allow, I will choose the teaching objects based on personal preferences before starting to guide new nurses/interns. P	36(7.3)	49(9.9)	84(17.0)	118(23.8)	208(42.0)
8. The frequency with which I show impatience when new nurses/interns perform poorly multiple times. N	20(4.0)	20(4.0)	65(13.1)	176(35.6)	214(43.2)
9. I have a high level of trust in the abilities of new nurses/interns in my daily work. P	107(21.6)	240(48.5)	129(26.1)	16(3.2)	3(0.6)
10. I allow new nurses/interns to have clinical exposure during guidance.	105(21.2)	257(51.9)	122(24.6)	10(2.0)	1(0.2)
11. The opportunities I can participate in hospital-assigned teaching method training. P	100(20.2)	138(27.9)	171(34.5)	73(14.7)	13(2.6)
12. Situations where mentoring work conflicts with clinical work.	Always	Often	Sometimes	Rarely	Hardly ever
	26(5.3)	40(8.1)	172(34.7)	182(36.8)	75(15.2)
	Hands-on ability	Communication ability	Work attitude	Learning ability	Creative spirit
13. The aspect for which I assign the highest weight in the comprehensive evaluation of the new nurses/interns I guide is:	118(23.8)	33(6.7)	284(57.4)	57(11.5)	3(0.6)
14. The aspect for which I assign the lowest weight in the comprehensive evaluation of the new nurses/interns I guide is:	49(9.9)	36(7.3)	43(8.7)	34(6.9)	333(67.3)

### Correlation analysis of motivation, attitude, and practice

Correlation analyses further showed that the motivation scores were positively correlated with attitude scores (*r* = 0.498, *P* < 0.001) and practice scores (*r* = 0.408, *P* = 0.001), and attitude scores were also positively correlated with practice scores (*r* = 0.554, *P* < 0.001) (Table 5).

**Table 5:** Correlation of scores on the dimensions of motivation, attitude and practice

	Motivation	Attitude	Practice
Motivation	1.000	0.498(*P*<0.001)	0.408(*P*<0.001)
Attitude	0.498(*P*<0.001)	1.000	0.554(*P*<0.001)
Practice	0.408(*P*<0.001)	0.554(*P*<0.001)	1.000

### Univariate and multivariate analyses of motivation, attitude, and practice

Compared to junior nursing mentors, the probability of higher motivation scores for intermediate and senior nursing mentors decreased by 36.2% (OR = 0.638, 95% CI: [0.426–0.956], *P* = 0.030). Compared to nursing mentors in Tertiary A hospitals, those in other hospitals had a 62.7% higher probability of having higher motivation scores (OR = 1.627, 95% CI: [1.054–2.511], *P* = 0.028).

The frequency of psychological care provided by the hospital resulted as a significant factor associated with nursing mentoring motivation, attitude, and practice, i.e., the higher the frequency of nursing mentors receiving psychological care from their hospital, the higher the probability of having a higher score. Compared to nursing mentors who did not participate in training, those who participated had a higher probability of having higher practice scores (OR = 2.908, 95% CI: [1.430, 5.913], *P* = 0.003). Compared to a higher frequency of job evaluations in hospitals, a lower frequency was a risk factor for nursing mentors having a higher probability of practice scores (“Often”: OR = 0.416, 95% CI: [0.244–0.709], *P* = 0.001 and “Sometimes”: OR = 0.346, 95% CI: [0.184–0.650], *P* = 0.001) **(**Table 6**).**

**Table 6:** Logistic regression analysis of the dimensions of motivation, attitude, and practice

Motivation (cut-off value: ≥29 /<29)		Univariate		Multivariate (forward method, *P*<0.1)	
	No.	OR(95%CI)	*P*	OR(95%CI)	*P*
**Age_adjusted**					
20–29 years	34/64	ref.			
30–39 years	181/340	1.004(0.588, 1.715)	0.987		
≥ 40 years	45/91	0.863(0.455, 1.637)	0.652		
**Gender**					
Male	4/10	0.596(0.166, 2.140)	0.428		
Female	256/485	ref.			
**Education_adjusted**					
Junior college and below	20/35	1.222(0.611, 2.447)	0.571		
Undergraduate and above	240/460	ref.			
**Professional title_adjusted**					
Junior (Nurse/Nurse Practitioner)	94/158	ref.		ref.	
Intermediate and Senior (Nurse Practitioner-in-Charge/Deputy Chief Nurse/Chief Nurse)	166/337	0.661(0.451, 0.969)	0.034	0.638(0.426, 0.956)	0.030
**Years of nursing experience**					
≤ 5 years	17/30	ref.			
6–10 years	71/145	0.734(0.332, 1.620)	0.444		
11–15 years	105/185	1.004(0.461, 2.186)	0.993		
16–20 years	46/96	0.704(0.308, 1.607)	0.404		
≥ 21 years	21/39	0.892(0.342, 2.325)	0.815		
**Grade of hospital_adjusted**					
Tertiary A	184/368	ref.		ref.	
Other	76/127	1.490(0.990, 2.244)	0.056	1.627(1.054, 2.511)	0.028
**Years of nursing supervision_adjusted**					
< 1 years	17/30	ref.			
1–2 years	23/46	0.765(0.303, 1.928)	0.570		
3–5 years	91/167	0.916(0.418, 2.005)	0.826		
6–10 years	84/155	0.905(0.411, 1.990)	0.803		
11–15 years	30/69	0.588(0.248, 1.397)	0.229		
≥ 15 years	15/28	0.882(0.313, 2.486)	0.813		
**Number of persons mentored**					
1–2	19/39	ref.			
3–5	46/82	1.345(0.626, 2.889)	0.447		
6–10	49/93	1.172(0.555, 2.477)	0.677		
≥ 11	146/281	1.138(0.582, 2.225)	0.705		
**Pathways to becoming a mentor_adjusted**					
Self-application	42/61	ref.			
Organization arrangement	210/420	0.452(0.255, 0.804)	0.007		
Other	8/14	0.603(0.184, 1.981)	0.405		
**Training attending**					
Have not attended training	18/56	ref.			
Did attend training	225/403	2.669(1.473, 4.835)	0.001		
Don’t remember	17/36	1.889(0.798, 4.472)	0.148		
**Salary subsidies granted by hospital**					
Always	54/86	ref.			
Often	45/64	1.404(0.703, 2.804)	0.337		
Sometimes	47/76	0.960(0.508, 1.815)	0.901		
Rarely	36/70	0.627(0.331, 1.191)	0.154		
Hardly ever	78/199	0.382(0.227, 0.644)	<0.001		
**Degree of hospital financial support**					
Adequate	79/115	ref.			
Much	48/71	0.951(0.504, 1.793)	0.877		
Ordinary	82/175	0.402(0.245, 0.658)	<0.001		
Very little	23/48	0.419(0.210, 0.836)	0.014		
Hardly ever	28/86	0.220(0.121, 0.400)	<0.001		
**Frequency of psychological care performed by hospital**					
Always	87/126	ref.		ref.	
Often	83/138	0.676(0.407, 1.125)	0.132	0.677(0.405, 1.132)	0.137
Sometimes	61/144	0.329(0.199, 0.544)	<0.001	0.316(0.190, 0.526)	<0.001
Rarely	26/61	0.333(0.177, 0.627)	0.001	0.311(0.163, 0.591)	<0.001
Hardly ever	3/26	0.058(0.017, 0.206)	<0.001	0.057(0.016, 0.203)	<0.001
**Frequency of hospital to understanding issues in mentoring**					
Always	99/173	ref.			
Often	82/172	0.681(0.445, 1.041)	0.076		
Sometimes	54/98	0.917(0.557, 1.511)	0.735		
Rarely	17/36	0.669(0.325, 1.374)	0.274		
Hardly ever	8/16	0.747(0.268, 2.084)	0.578		
**Frequency of job evaluation in hospital**					
Always	90/136	ref.			
Often	119/232	0.538(0.347, 0.835)	0.006		
Sometimes	45/110	0.354(0.210, 0.595)	<0.001		
Rarely	4/10	0.341(0.092, 1.268)	0.108		
Hardly ever	2/7	0.204(0.038, 1.095)	0.064		
**Attitude (cut-off value: ≥87 /<87)**		**Univariate**	**Multivariate (forward method**, ***P*<0.1)**	**Attitude (cut-off value: ≥87 /<87)**	
**Age_adjusted**		**OR(95%CI)**	***P***		
20–29 years	25/64	ref.			
30–39 years	176/340	1.674(0.970, 2.888)	0.064		
≥ 40 years	50/91	1.902(0.993, 3.645)	0.053		
**Gender**					
Male	4/10	0.642(0.179, 2.305)	0.497		
Female	247/485	ref.			
**Education_adjusted**					
Junior college and below	19/35	1.167(0.586, 2.326)	0.661		
Undergraduate and above	232/460	ref.			
**Professional title_adjusted**					
Junior (Nurse/Nurse Practitioner)	73/158	ref.			
Intermediate and Senior (Nurse Practitioner-in-Charge/Deputy Chief Nurse/Chief Nurse)	178/337	1.304(0.892, 1.904)	0.170		
**Years of nursing experience**					
≤ 5 years	12/30	ref.		ref.	
6–10 years	58/145	1.000(0.448, 2.231)	1.000	0.961(0.417, 2.216)	0.926
11–15 years	109/185	2.151(0.979, 4.726)	0.056	2.043(0.899, 4.643)	0.088
16–20 years	48/96	1.500(0.652, 3.450)	0.340	1.345(0.565, 3.202)	0.503
≥ 21 years	24/39	2.400(0.906, 6.360)	0.078	2.241(0.818, 6.134)	0.116
**Grade of hospital_adjusted**					
Tertiary A	191/368	ref.			
Other	60/127	0.830(0.554, 1.243)	0.366		
**Years of nursing supervision_adjusted**					
< 1 years	16/30	ref.			
1–2 years	21/46	0.735(0.292, 1.849)	0.513		
3–5 years	76/167	0.731(0.335, 1.593)	0.430		
6–10 years	84/155	1.035(0.473, 2.267)	0.931		
11–15 years	35/69	0.901(0.382, 2.126)	0.811		
≥ 15 years	19/28	1.847(0.634, 5.382)	0.261		
**Number of persons mentored**					
1–2	18/39	ref.			
3–5	36/82	0.913(0.425, 1.964)	0.816		
6–10	41/93	0.920(0.434, 1.949)	0.827		
≥ 11	156/281	1.456(0.743, 2.851)	0.273		
**Pathways to becoming a mentor_adjusted**					
Self-application	34/61	ref.			
Organization arrangement	211/420	0.802(0.467, 1.376)	0.423		
Other	6/14	0.596(0.184, 1.925)	0.387		
**Training attending**					
Have not attended training	18/56	ref.			
Did attend training	215/403	2.414(1.333, 4.373)	0.004		
Don’t remember	18/36	2.111(0.892, 4.994)	0.089		
**Salary subsidies granted by hospital**					
Always	58/86	ref.			
Often	36/64	0.621(0.318, 1.212)	0.162		
Sometimes	38/76	0.483(0.255, 0.913)	0.025		
Rarely	36/70	0.511(0.267, 0.980)	0.043		
Hardly ever	83/199	0.345(0.203, 0.588)	<0.001		
**Degree of hospital financial support**					
Adequate	74/115	ref.			
Much	37/71	0.603(0.330, 1.101)	0.100		
Ordinary	77/175	0.435(0.268, 0.707)	0.001		
Very little	26/48	0.655(0.330, 1.298)	0.225		
Hardly ever	37/86	0.418(0.236, 0.742)	0.003		
**Frequency of psychological care performed by hospital**					
Always	90/126	ref.		ref.	
Often	79/138	0.536(0.321, 0.895)	0.017	0.535(0.317, 0.901)	0.019
Sometimes	55/144	0.247(0.148, 0.413)	<0.001	0.252(0.149, 0.424)	<0.001
Rarely	4/10	0.388(0.104, 1.440)	0.157		
Hardly ever	2/7	0.233(0.043, 1.243)	0.088		
**Practice (cut-off value: ≥41 /<41)**		**Univariate**	**Multivariate (forward method,** ***P*** **<0.1)**	**Multivariate (forward method,** ***P*** **<0.25)**	
**Age_adjusted**					
20–29 years	29/64	ref.			
30–39 years	183/340	1.407(0.823, 2.405)	0.212		
≥ 40 years	46/91	1.234(0.650, 2.342)	0.521		
**Gender**					
Male	3/10	0.387(0.099, 1.512)	0.172		
Female	255/485	ref.			
**Education_adjusted**					
Junior college and below	19/35	1.098(0.551, 2.189)	0.790		
Undergraduate and above	239/460	ref.			
**Professional title_adjusted**					
Junior (Nurse/Nurse Practitioner)	82/158	ref.			
Intermediate and Senior (Nurse Practitioner-in-Charge/Deputy Chief Nurse/Chief Nurse)	176/337	1.013(0.694, 1.479)	0.946		
**Years of nursing experience**					
≤ 5 years	15/30	ref.			
6–10 years	65/145	0.812(0.370, 1.785)	0.605		
11–15 years	105/185	1.312(0.606, 2.842)	0.490		
16–20 years	53/96	1.233(0.542, 2.801)	0.618		
≥ 21 years	20/39	1.053(0.406, 2.727)	0.916		
**Grade of hospital_adjusted**					
Tertiary A	186/368	ref.			
Other	72/127	1.281(0.853, 1.923)	0.232		
**Years of nursing supervision_adjusted**					
< 1 years	17/30	ref.			
1–2 years	23/46	0.765(0.303, 1.928)	0.570		
3–5 years	84/167	0.774(0.354, 1.694)	0.521		
6–10 years	80/155	0.816(0.371, 1.793)	0.612		
11–15 years	37/69	0.884(0.373, 2.096)	0.780		
≥ 15 years	17/28	1.182(0.415, 3.368)	0.755		
**Number of persons mentored**					
1–2	19/39	ref.			
3–5	40/82	1.003(0.468, 2.149)	0.995		
6–10	48/93	1.123(0.531, 2.372)	0.762		
≥ 11	151/281	1.223(0.625, 2.390)	0.557		
**Pathways to becoming a mentor_adjusted**					
Self application	38/61	ref.			
Organization arrangement	213/420	0.623(0.359, 1.082)	0.093		
Other	7/14	0.605(0.188, 1.947)	0.400		
**Training attending**					
Have not attended training	13/56	ref.		ref.	
Did attend training	229/403	4.353(2.271, 8.346)	<0.001	2.908(1.430, 5.913)	0.003
Don’t remember	16/36	2.646(1.072, 6.534)	0.035	2.559(0.968, 6.765)	0.058
**Salary subsidies granted by hospital**					
Always	58/86	ref.			
Often	37/64	0.662(0.338, 1.293)	0.227		
Sometimes	39/76	0.509(0.269, 0.962)	0.038		
Rarely	38/70	0.573(0.299, 1.100)	0.094		
Hardly ever	86/199	0.367(0.216, 0.625)	<0.001		
**Degree of hospital financial support**					
Adequate	79/115	ref.			
Much	36/71	0.469(0.255, 0.862)	0.015		
Ordinary	91/175	0.494(0.301, 0.808)	0.005		
Very little	18/48	0.273(0.135, 0.553)	<0.001		
Hardly ever	34/86	0.298(0.166, 0.535)	<0.001		
**Frequency of psychological care performed by hospital**					
Always	96/126	ref.		ref.	
Often	74/138	0.361(0.213, 0.613)	<0.001	0.582(0.323, 1.050)	0.072
Sometimes	65/144	0.257(0.152, 0.435)	<0.001	0.473(0.259, 0.863)	0.015
Rarely	17/61	0.121(0.060, 0.242)	<0.001	0.226(0.106, 0.483)	<0.001
Hardly ever	6/26	0.094(0.034, 0.255)	<0.001	0.185(0.062, 0.549)	0.002
**Frequency of hospital to understanding issues in mentoring**					
Always	121/173	ref.			
Often	84/172	0.410(0.264, 0.638)	<0.001		
Sometimes	33/98	0.218(0.128, 0.371)	<0.001		
Rarely	14/36	0.273(0.130, 0.576)	0.001		
Hardly ever	6/16	0.258(0.089, 0.747)	0.012		
**Frequency of job evaluation in hospital**					
Always	102/136	ref.		ref.	
Often	111/232	0.306(0.192, 0.487)	<0.001	0.416(0.244, 0.709)	0.001
Sometimes	41/110	0.198(0.115, 0.343)	<0.001	0.346(0.184, 0.650)	0.001
Rarely	2/10	0.083(0.017, 0.412)	0.002	0.436(0.076, 2.480)	0.349
Hardly ever	2/7	0.133(0.025, 0.719)	0.019	0.420(0.067, 2.645)	0.356

## Discussion

The present study revealed that clinical nursing mentors in Zhejiang province, a multi-center research location in China’s economically advanced eastern coastal region, had adequate motivation, positive attitude, proactive practice towards mentoring, and other associated factors. Our findings emphasize the importance of enhancing clinical nursing mentorship. By prioritizing mentor training, fostering a supportive environment with consistent psychological care, and promoting structured mentorship activities, nursing mentors’ motivation, attitude, and practice could be significantly improved.

Our results showed that clinical nursing mentors had generally positive motivation, attitude, and proactive practice levels, which is consistent with the findings of the previous study, revealing that clinical mentors had high levels of motivation and a positive attitude towards their mentees that fostered a supportive mentorship environment [11]. Several factors were found to influence the motivation, attitude, and practice of nursing mentors, including the mentor’s professional title, years of experience, the number of apprentices they guide, and how they became mentors. Additionally, participation in training, compensation subsidies from hospitals, financial support, psychological care, and awareness of mentoring issues by hospitals all shaped mentors’ motivation, attitude, and practice. Our findings are consistent with previous studies, emphasizing the importance of institutional support and mentor training programs in improving mentorship quality [12, 13].

Our results indicated that the probability of higher motivation scores among nursing mentors varied depending on their experience and the type of hospital in which they worked. Moreover, it is essential to recognize that the probability of achieving higher motivation scores was not consistent across different mentorship experience levels. Mentors with more experience may need specific interventions to maintain their motivation. The frequency of psychological care provided by hospitals was a crucial factor affecting nursing mentoring motivation, attitude, and practice scores. Moreover, participation in training significantly affected the practice scores of nursing mentors, just as the assessment frequency, which underlines the importance of providing mentors with the necessary support and training to enhance their practice, thus increasing the quality of nursing mentorship [14]. In order to improve clinical practice in nursing mentorship, it is crucial to recognize the significance of mentor experience, the role of hospitals, and the need for ongoing training and support [15, 16]. By implementing targeted initiatives, healthcare institutions can enhance the quality of clinical nursing mentorship, ultimately contributing to developing a skilled and motivated nursing workforce [17, 18].

The responses in the motivation dimension reveal the nurses’ attitudes towards mentoring new nurses/interns. Nurses have diverse motivations for mentoring; for instance, some saw mentoring as a means of advancing their careers, while others did it out of a sense of duty to the organization. One objective way to improve clinical practice could be to encourage nurses to view mentoring as an opportunity for professional growth rather than just a responsibility [19, 20]. This shift in mindset may lead to more engaged and effective mentoring, ultimately benefiting both mentors and mentees.

The attitude dimension clarifies how the nursing mentor system is perceived and affects work-life balance. Many respondents believed mentoring could help coordinate scheduling, work-family balance, and relationships between new nurses/interns and colleagues. However, they also had concerns about the time and effort required for mentoring. An objective statement to enhance clinical practice is to emphasize the potential benefits of mentoring, such as the development of relationships and the discovery of talent [21, 22]. Institutions could provide resources and support to help mentors effectively manage their time, thus ensuring that mentoring does not become overly burdensome [23, 24].

The practice dimension focuses on the actions and behaviors of mentors during the mentoring process. Respondents were willing to adjust their guidance methods, seek help when needed, and continuously enhance their nursing knowledge. However, some expressed occasional impatience, and conflicts between mentoring and clinical work were not uncommon. The comprehensive evaluation of new nurses/interns involves various aspects, including hands-on ability, communication ability, work attitude, learning ability, and creative spirit. The weight assigned to these aspects varied among mentors. In order to improve clinical practice, it is essential to provide mentors with training and resources to manage mentoring challenges while maintaining their own clinical responsibilities [25]. Additionally, emphasizing the importance of patience, constructive guidance, and clinical exposure for new nurses/interns can promote a more positive mentoring experience [26, 27]. It is also recommended that standardized evaluation criteria be developed based on a balanced assessment of these attributes [28].

Our results revealed significant positive correlations among the dimensions of motivation, attitude, and practice scores in nursing mentorship. Specifically, nurses who exhibited higher levels of motivation were more likely to maintain positive attitudes toward mentoring, and those with positive attitudes were more likely to demonstrate effective mentoring practices. This interconnectedness underscores the importance of nurturing motivation among mentors, as it serves as a catalyst for fostering constructive attitudes and productive mentorship practices [29, 30]. Healthcare institutions should recognize the holistic nature of mentorship and aim to create an environment that encourages and sustains motivation while also providing mentorship training and support to enhance attitudes and practices [31, 32].

By examining nursing mentors’ motivations, attitudes, and practices, this study furthered the understanding of clinical nursing mentorship in Zhejiang Province, China. Its focus on an economically developed region provided insights into how regional factors influence mentorship, a topic that has not been widely covered in previous research. The present study also highlighted the importance of mentor training, psychological care, and structured mentorship activities, offering practical suggestions for improving mentorship programs.

The present study has some limitations, including its regional focus and sampling method. The reported findings, based solely on data from Zhejiang Province, may not apply to areas with different economic and cultural settings. Also, the sample that was drawn from 30 hospitals in one province might not fully represent all nursing mentors, potentially affecting the generalizability of the results. Therefore, while the study provides valuable regional insights, its applicability to other contexts is limited. Future research could benefit from a broader geographical range and a more diverse sampling to better represent the experience of nursing mentors.

Clinical nursing mentors had adequate motivation, positive attitudes and proactive practice towards mentoring and associated factors. Our results underscore several key recommendations for enhancing clinical nursing mentorship in practice. First, institutions should prioritize mentorship training programs to bolster clinical nursing mentors’ motivation, attitude, and practice, especially for those in intermediate and senior roles. Additionally, fostering a supportive institutional environment where psychological care is consistently provided can positively impact nursing mentors’ motivation, attitude, and practice. Moreover, addressing concerns and providing structured mentorship activities for individuals with lower probability ratings can significantly improve the quality and efficacy of mentorship in nursing.

### Electronic supplementary material

Below is the link to the electronic supplementary material.

**Supplementary Material 1:** Questionnaire

## Data Availability

No datasets were generated or analysed during the current study.
