# Supplementary material for: Clinical nursing mentors’ motivation, attitude, and practice for mentoring and factors associated with them
Source: BMC Nurs. 2024 Jan 30;23:76. doi: 10.1186/s12912-024-01757-8 (PMC10826088; doi:10.1186/s12912-024-01757-8)
Supplement: Supplementary file 1 — Supplementary Material 1: Questionnaire [file 12912_2024_1757_MOESM1_ESM.docx]

Dear participants,

We are researchers from Ningbo Institute of Health Professions and Technology, and we sincerely invite you to participate in our research project. This study aims to understand the clinical nursing mentors' motivation for mentoring and factors associated with it, which will contribute to improving the effectiveness of the nursing mentor system. It holds significant practical importance for the development of nursing human resources and the nursing profession. Your participation in this study is entirely voluntary. If you agree to participate, please refer to the following instructions.

1. Please complete the questionnaire. There are no right or wrong answers; you only need to provide responses based on your actual experiences. If you have any questions during the process, feel free to reach out to us, and please submit the completed questionnaire in a timely manner.
2. This study is a simple questionnaire survey and will not cause any harm to your physical or psychological well-being. However, it may involve some personal information such as your gender and age. Please rest assured that we will strictly maintain confidentiality and will not disclose your information.
3. As a participant, you can always stay informed about the information and progress related to this study. If you decide to withdraw from the study, please let us know, and your data will not be included in the research results.

Finally, we sincerely thank you for taking the time to support our scientific research amid your busy schedule!

□I have been informed and agreed to the use of the collected data for scientific research.

**Clinical Nursing Mentoring Motivation and Factors Associated with It**

In this study, "clinical nursing mentor" refers to a nurse who holds a nursing license, has the ability, and has previously undertaken the task of guiding novice nurses, such as newly hired nurses or nursing interns, further trained nurses, or nurses undergoing standardized training. Typically, during rotations in a particular department, the relationship between newly hired nurses or nursing interns and their clinical nursing mentor remains relatively stable. Various hospitals may use different terms to refer to "clinical nursing mentors," such as clinical teaching instructors, nursing mentors, masters, instructors, chief instructors, and others. All these terms fall within the conceptual scope of "clinical nursing mentor" in this study.

**Have you ever served or currently serve as a clinical nursing mentor?**

**(A) Yes; (B) No**

**Part I Basic Information**

1. Your age：

a.20-29 years

b.30-39 years

c.40-49 years

d.50 years and above

2.Your gender：

a.Male b.Female

3.Your education：

a.Junior college and below

b.Undergraduate

c.Postgraduate

d.PhD and above

4.Your professional title：

a.Nurse

b.Nurse Practitioner

c. Nurse Practitioner-in-Charge

d. Deputy Chief Nurse

e. Chief Nurse

5.Your years of nursing experience:

a. 5 years and below

b. 6-10 years

c.11-15 years

d.16-20 years

e.21 years and above

6.Your working institution is：__________________

7.Your hospital grade is:

a.Tertiary A

b.Tertiary B

c.Secondary A

d.Secondary B

e.Primary A

f.Primary B

8.How many years have you been a nursing mentor (since mentoring your first new or practice nurse)：

a. Less than 1 year

b.1-2 years

c.3-5 years

d. 6-10 years

e.11-15 years

f.16-20 years

g. 21 years and above

9.How many new/practising nurses have you mentored in total (including advanced practice nurses, interns, regulation nurses, etc.):

a.No mentoring experience yet

b.1-2

c.3-5

d.6-10

e.11 and above

10.Your pathway to becoming a clinical nursing mentor is:

a.Self application

b.Organization arrangement

c.Recommended by others

d.Other

11.Your department is:

The following is converted into a first-person account

12.Did you attend relevant teacher qualification training before becoming a clinical nursing mentors

a.Have not attended training b.Did attend training c.Don't remember

13.My hospital subsidises the salary of the clinical nursing mentors.

a.Always b.Often c.Sometimes d.Rarely e.Hardly ever

14.The degree of financial support for clinical nursing mentor in my hospital.

a.Adequate b.Much c.General d.Very little e.Hardly ever

15.Frequency of psychological care for clinical nursing mentors performed by my hospital.

a.Always b.Often c.Sometimes d.Rarely e.Hardly ever

16.Frequency of my hospital to understanding issues in mentoring.

a.Always b.Often c.Sometimes d.Rarely e.Hardly ever

17.The frequency of evaluation of clinical nursing mentors' work at my hospital.

a.Always b.Often c.Sometimes d.Rarely e.Hardly ever

**Part II**  mentors' motivation for mentoring

1. I participate in guiding new nurses/interns to accumulate opportunities for promotion.

a.Strongly agree b.Agree c.Unsure d.Disagree e.Strongly disagree

2. Participating in guiding new nurses/interns is a way for me to earn more financial rewards.

a.Strongly agree b.Agree c.Unsure d.Disagree e.Strongly disagree

3. I guide new nurses/interns because it's an organizational assignment, and I have no choice.

a.Strongly agree b.Agree c.Unsure d.Disagree e.Strongly disagree

4. When guiding new nurses/interns, I prefer to see them as partners in our work rather than in a mentor-apprentice relationship.

a.Strongly agree b.Agree c.Unsure d.Disagree e.Strongly disagree

5. I guide new nurses/interns to help them become competent in nursing as quickly as possible.a.Strongly agree b.Agree c.Unsure d.Disagree e.Strongly disagree

6. I participate in guiding new nurses/interns to study the patterns of nursing talent development.

a.Strongly agree b.Agree c.Unsure d.Disagree e.Strongly disagree

7. I guide new nurses/interns to pass on the spirit of the nursing profession.

a.Strongly agree b.Agree c.Unsure d.Disagree e.Strongly disagree

8. I guide new nurses/interns to make them fall in love with the noble profession of nursing.

a.Strongly agree b.Agree c.Unsure d.Disagree e.Strongly disagree

**Part III Mentors' attitude to mentoring**

1.To what extent can nursing mentor system address the following issues in job satisfaction of new nurses/interns.

1.1 The nursing mentor system can help coordinate issues related to the scheduling of new nurses/interns.

a.Strongly agree b.Agree c.Unsure d.Disagree e.Strongly disagree

1.2 The nursing mentor system can help balance the work and family life of new nurses/interns

a.Strongly agree b.Agree c.Unsure d.Disagree e.Strongly disagree

1.3 The nursing mentor system can help coordinate relationships between new nurses/interns and colleagues.

a.Strongly agree b.Agree c.Unsure d.Disagree e.Strongly disagree

2. Although participating in guiding new nurses, the growth of new nurses/interns is beneficial to themselves, but it doesn't benefit me much.。

a.Strongly agree b.Agree c.Unsure d.Disagree e.Strongly disagree

3. Participating in guiding new nurses/interns takes up too much of my time.

a.Strongly agree b.Agree c.Unsure d.Disagree e.Strongly disagree

4. The reporting and documentation tasks during the guidance of new nurses/interns are cumbersome and affect my enthusiasm for mentoring.

a.Strongly agree b.Agree c.Unsure d.Disagree e.Strongly disagree

5. Participating in guiding new nurses/interns allows them to share some of my workload.

a.Strongly agree b.Agree c.Unsure d.Disagree e.Strongly disagree

6.Participating in guiding new nurses/interns is beneficial for building a good relationship with the nursing department (superiors).

a.Strongly agree b.Agree c.Unsure d.Disagree e.Strongly disagree

7. Participating in guiding new nurses/interns can help discover talent and build a broader network of relationships.

a.Strongly agree b.Agree c.Unsure d.Disagree e.Strongly disagree

8. Guiding new nurses/interns pushes me to continue learning.a.Strongly agree b.Agree c.Unsure d.Disagree e.Strongly disagree

9. Able to learning new ideas and concepts from young nurses is very helpful to me.

a.Strongly agree b.Agree c.Unsure d.Disagree e.Strongly disagree

10. Participating in guiding new nurses/interns is helpful for me to learn new knowledge and experiences from other mentors.

a.Strongly agree b.Agree c.Unsure d.Disagree e.Strongly disagree

11. I also received help from others when I was newly employed, so I am willing to actively mentor new nurses/interns.

a.Strongly agree b.Agree c.Unsure d.Disagree e.Strongly disagree

12. Participating in guiding new nurses/interns can earn me recognition and approval from colleagues.

a.Strongly agree b.Agree c.Unsure d.Disagree e.Strongly disagree

13. Helping new nurses/interns grow makes me feel very happy.

a.Strongly agree b.Agree c.Unsure d.Disagree e.Strongly disagree

14. Watching new nurses/interns grow under my guidance makes me feel my own value.

a.Strongly agree b.Agree c.Unsure d.Disagree e.Strongly disagree

15. New mentors should receive training on educational and teaching abilities.

a.Strongly agree b.Agree c.Unsure d.Disagree e.Strongly disagree

16. Nurses should only take on mentorship tasks after undergoing a comprehensive assessment.

a.Strongly agree b.Agree c.Unsure d.Disagree e.Strongly disagree

17. If given the opportunity to become an excellent mentor, I will guide new nurses/interns more.

a.Strongly agree b.Agree c.Unsure d.Disagree e.Strongly disagree

18. The organization has nurtured me, and I should also nurture new talents for the organization.

a.Strongly agree b.Agree c.Unsure d.Disagree e.Strongly disagree

19. Serving as a mentor allows me to share my experiences and lessons with youngsters, helping them avoid unnecessary mistakes.

a.Strongly agree b.Agree c.Unsure d.Disagree e.Strongly disagree

20. I care deeply about new nurses/interns because I have had similar experiences.

a.Strongly agree b.Agree c.Unsure d.Disagree e.Strongly disagree

**Part IV Mentors' practice on mentoring**

1. I will adjust the guidance methods for new nurses/interns with different personalities.

a.Always b.Often c.Sometimes d.Rarely e.Hardly ever

2. I care about the learning, work, and life issues encountered by new nurses/interns.

a.Once per day b.Twice a week c.Once per week d.Once per 2 weeks e.Once per month

3. I regularly summarize methods and organize records during the guidance of new nurses/interns.

a.Always b.Often c.Sometimes d.Rarely e.Hardly ever

4. When I encounter problems that cannot be resolved during the guidance of new nurses/interns, I seek help from the department or nursing department.

a.Always b.Often c.Sometimes d.Rarely e.Hardly ever

5. I continuously enhance my nursing knowledge and comprehensive abilities during the guidance of new nurses/interns.

a.Always b.Often c.Sometimes d.Rarely e.Hardly ever

6. The frequency with which I consider giving up during the process of guiding new nurses/interns.

a.Always b.Often c.Sometimes d.Rarely e.Hardly ever

7. If conditions allow, I will choose the teaching objects based on personal preferences before starting to guide new nurses/interns.

a.Always b.Often c.Sometimes d.Rarely e.Hardly ever

8. The frequency with which I show impatience when new nurses/interns perform poorly multiple times.

a.Always b.Often c.Sometimes d.Rarely e.Hardly ever

9. I have a high level of trust in the abilities of new nurses/interns in my daily work.

a.Always b.Often c.Sometimes d.Rarely e.Hardly ever

10. I allow new nurses/interns to have clinical exposure during the guidance process.

a.Always b.Often c.Sometimes d.Rarely e.Hardly ever

11. The opportunities I can participate in hospital-assigned teaching method training.

a.Always b.Often c.Sometimes d.Rarely e.Hardly ever

12. Situations where mentoring work conflicts with clinical work.

a.Always b.Often c.Sometimes d.Rarely e.Hardly ever

13.The aspect for which I assign the highest weight in the comprehensive evaluation of the new nurses/interns I guide is:

a.Hands-on ability b.Communication ability c.Work attitude d.Learning ability e.Creative spirit

14.The aspect for which I assign the lowest weight in the comprehensive evaluation of the new nurses/interns I guide is:

a.Hands-on ability b.Communication ability c.Work attitude d.Learning ability e.Creative spirit
